# Dimensions of reading: a study of the beliefs of language and literature preservice teachers

**DOI:** 10.3389/fpsyg.2023.1284539

**Published:** 2023-10-04

**Authors:** Carolina González Ramírez, Enzo Pescara Vásquez

**Affiliations:** Departamento de Didáctica y Prácticas, Instituto de Literatura y Ciencias del Lenguaje, Facultad de Filosofía y Educación, Pontificia Universidad Católica de Valparaíso, Viña del Mar, Chile

**Keywords:** reading, reading habit, teacher education, literature, beliefs

## Abstract

This study aims to analyze the beliefs that future Language and Literature teachers hold regarding reading. This work is part of a broader research endeavor focused on the reading habits and practices of teachers in training and their role as prospective mediators since the way in which they perceive reading significantly impacts the mediation processes they undertake in their teaching practices to cultivate readers. To achieve these objectives, a multiple case study is conducted, involving interviews with 1st-year students (*n* = 15), 3rd-year students (*n* = 15), and 5th-year students (*n* = 15) enrolled in Language Pedagogy programs across three universities affiliated with the Chilean Council of Rectors. For data analysis, a content analysis approach is employed, supported by NVivo 12. The findings reveal that beliefs about reading primarily fall into two dimensions: academic and personal, with the former exhibiting clearer definition and characterization. This can be attributed to the influence of the disciplines integrated into their education, namely literature and linguistics. In conclusion, it is imperative to address the social dimension of reading during the initial teacher education program, as this aspect is not emphasized by preservice teachers, despite its pivotal role in shaping their identity as reading mediators within the context of their teaching practice.

## 1. Introduction

Reading is a complex construct that exhibits various conceptualizations depending on the disciplinary fields from which it is approached as a subject of study (Maina and Papalini, 2021) and also on the reading modes adopted, according to each reader’s experiences (Cuesta, 2006; Munita, 2016). According to Lluch and Zayas (2015), reading is currently conceived as an activity that demands the cultivation of skills in accessing and selecting information based on reading objectives, in addition to the ability to interpret texts. Alternatively, it can be regarded as the capacity to reflect upon what is read, contingent upon the social context within which one interacts. The foregoing is complemented by the assertions of Elche et al. (2019), who state that reading serves as the foundation through which learning is acquired. Thus, reading would be a tool for literacy and, consequently, it would contribute to the acquisition of knowledge (Trigos-Carrillo, 2019). On the other hand, it is considered an interactive process, mainly due to the dynamic nature of reading, where the reader would have an active role, as it is required going beyond mere decoding of the presented written content and implementing comprehension skills (Asselin, 2000). The foregoing is part of an academic dimension of reading, from which such activity, guided by specific objectives, would enable the reader to perform in various learning situations. Meanwhile, at a personal level, according to Lluch and Zayas (2015), reading is oriented towards satisfying an individual’s specific interests and maintaining personal relationships with others. It is within this context that reading for pleasure is situated. It refers to reading as a social practice (Margallo, 2012) and which, by definition, should be free and autonomous, as it takes place in leisure time and, simultaneously, it enhances various cognitive abilities that will contribute to one’s professional development (Sánchez-Chévez, 2012).

Given that our field of study falls within the realm of initial teacher education, it is deemed necessary to refer to studies associated with the beliefs held by both in-service and preservice educators regarding reading and its connection to this construct. This is understood in light of the fact that beliefs consist of cognitive propositions that are not necessarily structured and are derived from a personal dimension (Cambra et al., 2000). Thus, as pointed out by Jiménez et al. (2014), at the level of beliefs, individuals tend to pragmatically use theory to interpret situations and plan their behaviors. This, to some extent, explains why teachers’ beliefs about reading are pertinent, as these shape their practice as mediators of reading.

Building upon this notion, an investigative panorama is presented, enabling an understanding of the beliefs held by future Language and Literature teachers regarding this construct. However, it is important to note that this review is constrained, given that studies in this vein with future secondary Language and Literature teachers are scarce. Hence, reference will be made to research related to in-service and preservice teachers in primary and early childhood education to augment the theoretical framework.

In regard to service teachers, it is relevant to mention the insights put forth by Errázuriz et al. (2020), who indicate that diverse evidence has emerged concerning the impact of teachers’ beliefs and reading habits on their teaching practices which are related to fostering reading habits in the classroom. This phenomenon can be explained by the fact that research on teacher perceptions is grounded in the notion that a teacher’s instructional performance is conditioned by the representations they hold about the object of teaching and learning (Pozo et al., 2006; Jiménez et al., 2014). In this vein, the study conducted by Munita (2016) stands out, as it examines the relationship that female primary school teachers establish between their reading profiles, belief systems regarding literary reading, and their approach to literature in the school setting. In this work, the author underscores the significance of teachers’ beliefs in their instructional practices, since their conception of reading and literature determines their teaching practice, the formative objectives they pursue, and their role as mediators within the classroom context. While individual differences may exist, all concur that the primary goal is to cultivate engaged readers. Taking this point into consideration, it becomes imperative to have well-prepared teachers to mediate and facilitate the reading process, who are actively engaged in the construction of reading pathways and capable of guiding diverse disciplinary readings. This necessary endeavor extends beyond the conventional or general pedagogical scope, as it is often the case with primary education teachers (Errázuriz et al., 2022). To this end, it is essential that teachers serve as role models for reading and take on the task of teaching how to read literary works. However, it has been noted that in-service teachers are not always ideal reading models due to a deficiency in the frequency of personal reading engagements (Díaz-Díaz et al., 2022).

In the realm of initial teacher education, studies concerning the relationship that preservice teachers maintain with reading reveal its inadequacy. This is affirmed by international research wherein a significant percentage of future educators exhibit reading profiles indicative of weak reading habits, as well as a limited interest in autonomous reading (Díaz-Díaz et al., 2022) alongside minimally developed beliefs about reading (Shaw and Mahlios, 2011). In the study conducted by Larrañaga and Yubero (2019), 861 students enrolled in the Bachelor’s program for Early Childhood and Primary Education were surveyed to investigate their reading behavior and engagement as it is expected they develop competencies that enable them to train readers. In this study, 40.4% of the participants reported being occasional readers, which undoubtedly raises concerns and invites reflection. If their role is to nurture regular readers, they would lack a habit strong enough to impart to their potential students, and they also lack a clear understanding of what reading signifies for them. A similar situation was reported by Granado and Puig (2015), who indicated that 43.2% of prospective teachers identify themselves as weak readers and lack a clear self-perception as readers. Lastly, in the study by Vera Valencia (2017), 32.3% of future educators stated that they never or rarely read for pleasure, underscoring that reading is not part of their daily activities.

Meanwhile, the study by Muñoz et al. (2018) presents a similar landscape, as they conclude that future teachers in primary and early childhood education from five Chilean universities do not exhibit reading habits, nor do they demonstrate engagement with reading either academically or personally. Regarding Language and Literature teachers, Asfura and Real (2019) who investigated the reading aspect of teachers in this group upon their graduation, portrayed a reading profile that distinguishes between reading practices in the academic realm and personal space, based on three dimensions: the familial and social environment for reading, the place of reading in their daily lives, and literary reading. Based on the above, the authors argue that preservice teachers exhibit a developing reading habit, alongside, these teachers lack a significant relationship with literature. Similarly, the study by Merino et al. (2020) underscores the notion that these prospective educators lack a robust reading foundation upon entering higher education and that their affinity for reading increases as they progress in their pedagogy studies, thereby reaffirming the idea that this group of future teachers is also characterized by being low readers, and their relationship with reading is weak.

Regarding the beliefs of preservice teachers, about reading, it is pertinent to mention the study by González Ramírez et al. (2020), where it was reported that a group of prospective primary and early childhood education teachers primarily recognize three facets of reading: functional, cognitive, and social. The results demonstrate that future teachers define reading based on its functional facet (71%), followed by the cognitive facet (25%), and lastly, the social facet (4%). These results align with the findings by Álvarez-Álvarez and Diego-Mantecón (2019) and Míguez-Álvarez et al. (2023), where it is evident that future primary school teachers tend to approach reading and literature from an instrumental perspective, as they seldom engage in regular reading practices or derive personal enjoyment from it. This reflects that prospective teachers have not integrated reading as a regular practice but rather approach it as tools to accomplish specific tasks in their academic work. Their perspective does not extend towards their teaching practice; instead, their concept of reading is shaped by their experiences as students.

The results presented above are not promising and tend to be general in nature; thus, we believe that qualitative studies are needed to delve into the beliefs that future Language and Literature teachers hold about reading. As seen, teachers’ belief systems directly impact classroom performance, and since this group will serve as future mediators and will need to foster reading habits in secondary education, it is pertinent to understand how they conceptualize reading based on their beliefs. This is relevant because it offers insight into an essential aspect of their connection with reading.

## 2. Methods

This work, framed in educational research, is a qualitative investigation which applies an interpretative approach (Creswell, 2013). A multiple case study design was employed, following the guidelines outlined by Dörnyei (2007). This means that the study was extended to several cases, as the focus was not on isolated cases but rather on the collective as representative of a phenomenon (Dörnyei, 2007; Stake, 2007). It is important to note that this work is part of a larger, multi-stage research endeavor. It is organized into three phases: (1) the first one was contextualization, where we gained a deeper understanding of the Initial Teacher Education programs of the collaborating universities; (2) the second phase entailed inquiry with future teachers from three training programs, conducting interviews that were subsequently analyzed for the purposes of this study; finally, (3) the interpretation stage which involved analyzing the results obtained from the previous stages. Therefore, the primary aim of this study is to analyze the beliefs that prospective Language and Literature teachers hold about reading. The choice to begin with an examination of their beliefs about reading stems from the conviction that understanding their perspectives enables us to grasp their relationship with reading, besides it aids in the characterization of reader profiles.

### 2.1. Participants

The study includes a total of 45 students enrolled in first year (15), third year (15), and fifth year (15) of Pedagogy in Language programs from 3 universities belonging to the Chilean Council of University Rectors (CRUCH), as shown in Table 1. Their ages range between 18 and 25 years on average. The sample was collected through intentional, non-probabilistic convenience sampling (Sáez, 2017). Each participant agreed to take part voluntarily, following the relevant bioethical norms for conducting this research.

**Table 1:** Description of the participants.

Level	Gender/No. Participants	Semesters completed
1st year	Female: 11 Male: 4	1
3rd year	Female: 9 Male: 6	5
5th year	Female: 10 Male: 5	8

### 2.2. Data collection instruments

For data collection, semi-structured interviews (Kvale, 2011) were conducted with each participant. This technique was chosen as, according to Lluch and Zayas (2015), interviews provide access to information about participants’ reading and teaching habits, experiences, and practices. To achieve this, an interview protocol was designed after a thorough documentary and theoretical review. This instrument encompassed the data associated with the research and a script comprising 19 questions, which were grouped under the following themes: (1) reading habits, frequencies, and preferred genres; (2) practices of literary reading; and (3) conceptions about reading mediation both, in the school and initial training context. The first theme was addressed because it was of interest to know what and how much they read, both in their personal spaces and within academic settings, considering their self-perception as readers too, as it would allow to initially profile their reading habits. The second theme complements the previous one, as it allows us to delve into literary reading practices to explore their relationship with reading, as well as investigate where they typically engage in reading, seeking to identify which environments associated with their reading habits they frequently visit. Lastly, the third theme addresses two aspects: (a) their conceptions and beliefs about the mediation of literary reading in the school context, considering questions about reader training and the role of the literature teacher and (b) inquiry about their own initial training in pedagogy programs in this field, with the purpose of investigating how they perceive their training as reading mediators and whether the concept of mediation is effectively integrated into Initial Teacher Education programs.

### 2.3. Procedure

Between the months of June and November 2022, after adhering to ethical procedures and obtaining the relevant informed consents, participants were contacted via email to invite them to take part in the project and conduct semi-structured interviews. These interviews could be conducted via Zoom or in-person, depending on each participant’s availability. For the purposes of this study, a total of 45 interviews were transcribed and prepared for analysis. Each interview was assigned a code, and a pseudonym was used for each student. It’s important to note that the transcriptions are verbatim and faithfully represent the statements made by each participant.

### 2.4. Data analysis

In this study, a content analysis was employed following the guidelines outlined by Miles et al. (2014), who propose a two-cycle analysis. In the first coding cycle, an inventory of emerging codes was generated from the data gathered through interviews with prospective teachers. These codes were subsequently organized and systematized into dimensions and analytic categories during a second cycle, in order to construct a codebook. To achieve this, the researcher cross-validated the generated codes and categories as part of the analysis. The level of agreement between coders reached 98% for the total reviewed codes. Data analysis was facilitated through the use of NVivo 12 software.

## 3. Results

To present the findings, a preliminary exploration of reading beliefs will be provided. This initial examination highlights the assessment preservice teachers make of this construct. Subsequently, the focus will shift to the dimensions primarily elicited during the interviews when participants were queried about their perceptions of reading. This analysis encompasses both the academic dimension and the personal dimension, underscoring that this construct is linked to both matters of knowledge and to enjoyment and leisure activities primarily.

### 3.1. Evaluation of reading

Across all analyzed interviews, a positive evaluation towards reading was consistently observed. Participants converge on the notion that reading is an enjoyable activity. Moreover, they underscore the significance of reading for personal development. According to the perceptions of the prospective teachers, reading brings about changes and broadens perspectives. We highlight two representative statements that encapsulate these notions; the first from a first-year student and the second from a fifth-year student, indicating a shared sentiment within the participant group.

EU1_N1_02_Kira: I really like it because I feel that, this is how… getting to know different perspectives.

EU2_N5_04_Rodolfo: “But I stick with this idea that reading truly changes people, perhaps it broadens our perspective, tells us that there are things we were not seeing before.”

The metaphor of “broadening one’s perspective” alludes to the concept of gaining access to knowledge, a circumstance that would be facilitated by reading, as it enables an acquaintance with various perspectives and viewpoints, ultimately fostering a deeper learning experience. Nevertheless, this notion is limited as it refers to an instrumental approach to reading and literature, pointed out by Míguez-Álvarez et al. (2023). Most of the identified evaluations are primarily associated with the potential that reading offers for learning; therefore, it can be inferred that preservice teachers reading activity mainly occurs in academic environments. This highlights a weak connection with reading as a social practice linked to enjoyment in personal contexts (Álvarez-Álvarez and Diego-Mantecón, 2019; Díaz-Díaz et al., 2022).

### 3.2. Academic dimension

The academic dimension pertains to a perspective of reading centered on accessing knowledge and fostering learning. Prospective teachers draw upon their own experiences as students to articulate the significance of reading for them. It is noteworthy that the findings are consistent across the three groups, encompassing students in their 1st, 3rd, and 5th years of study. Among 5th-year students, references to disciplinary knowledge in relation to reading are more prevalent than among 1st-year students.

Within this dimension, two primary notions of reading are situated, conceptualized based on insights gleaned from the prospective teachers. Key associated concepts were considered in their analysis. The outcomes are presented in Table 2.

**Table 2:** Academic dimension of reading.

Academic dimension		
Categories	Conceptualization	Topics
Learning	Reading is understood as a tool for learning, as well as an epistemic ability that allows the subject to acquire knowledge and also build meaning from reading texts. This refers to a functional perspective.	Tool Ability Competence Interpretation
Knowledge	The underlying belief in this category refers to reading as a source of knowledge, which allows access to culture and different worldviews. Similarly, it is considered from a functional perspective	World view Access to knowledge Culture

Below are some excerpts from the interviews where references can be found that have contributed to the formulation of the conceptualization presented regarding learning:

EU1_N5_04_Fabiola: “To me, reading is an epistemic skill… Yes, for me, reading is first and foremost a skill or a competency that comprises many sub-skills contributing to learning, whether personal or for academic-disciplinary work.

EU1_N1_02_Kira: “For me, that’s it, learning, whatever one reads, to me, it’s learning.”

Regarding knowledge, the following statements are considered:

EU1_N1_02_Kira: “Above all, as I mentioned, it’s a contribution of culture… to people.”

EU2_N1_05_Carlos: “I really like it because I feel that, this is how… getting to know different perspectives.”

These notions are regarded as limited and inadequately developed in conceptual terms by the prospective teachers, which aligns with the findings of Shaw and Mahlios (2011). This emphasizes the necessity to expand the theoretical frameworks accessible to them, enabling the progression towards more extensively elaborated conceptualizations within disciplinary terms. As stated in previous sections, reading is a complex construct that presents different definitions (Maina and Papalini, 2021). Therefore, it is necessary for prospective teachers to have knowledge about this and be capable of reflecting on this idea so that the representations they construct are grounded in a conceptual framework, not solely based on their reading and literary experience.

### 3.3. Personal dimension of reading

The personal dimension of reading has been approached through the beliefs of the prospective teachers, wherein they affirm that reading is a leisure activity, a source of enjoyment, and relaxation. Table 3 presents the categories derived from their perspectives, the conceptualization of each, and the topics associated with them.

**Table 3:** Personal dimension of reading.

Personal dimension		
Categories	Conceptualization	Topics
Leisure activity	Reading is understood as a form of escape from reality, that is, a pastime that contributes to their recreation in their personal space, outside of the academic environment.	Escape or evasion entertainment disconnection Hobbie
Enjoyment	Reading is conceived as an instance in which fruition and aesthetic appreciation is experienced, mainly from literary reading.	Aesthetic enjoyment like

To comprehend the presented ideas, examples from the category labeled as “leisure activity” are provided, wherein reading becomes a moment of evasion or escape from reality:

EU1_N1_03_Josefa: just like some people have dedicated their entire lives to ballet, I’ve been reading my whole life… It’s been my hobby.

EU3_N3_05_Claudio: as a moment of disconnection where I can be with the book, and I know no one else is going to bother me and I know that what’s happening outside does not interest me at that moment.

Regarding enjoyment, the following example is relevant:

EU2_N5_02: with more literary reading, with juvenile classics, Narnia, Harry Potter to a certain extent… it’s a pleasure, for example, to imagine that story in one’s head and read it and think, “Oh! I’m enjoying this.”

In this way, it is possible to observe how the prospective teachers position reading as a pleasurable activity, one they enjoy and that allows them to escape from reality. However, it is noteworthy that the notion of disconnection or escape with which they associate reading is noticeable. This concept echoes the traditional notion of engaging in the act of reading a book. In this scenario, the reader sets aside their usual tasks to immerse themselves where the focus is on enjoyment through literary reading. This is evident in the discussed interventions, which explicitly refer to the book as a literary artifact.

## 4. Conclusion

In this preliminary study, it is possible to observe that the reading beliefs elicited from future Language and Literature teachers lack substantial conceptual elaboration, which can be attributed to their weak connection with reading (Larrañaga and Yubero, 2019). Nevertheless, a positive evaluation of reading was evident, emphasizing its significance in gaining access to cultural literacy. This is primarily due to the fact that reading continues to hold social prestige on a collective level, regardless of whether it is actively pursued as a regular practice (Díaz-Díaz et al., 2022).

Equally relevant to mention is that fifth-year students employ more abstract concepts to refer to reading compared to first-year students, who tend to use more generic concepts not directly associated with their field of study. This indicates that the education received while pursuing a pedagogy program has an impact on their beliefs regarding reading, although these beliefs still remain limited. Therefore, it is essential to explore potential approaches that empower future teachers to contemplate reading and their own personal reading habits. This should be supported by a reference framework that allows them to access various conceptualizations of reading from different spheres of knowledge, enabling them to construct definitions beyond their own experience. In this regard, it is considered relevant to emphasize the study of the social dimension of reading while they undergo initial teacher training programs. This is a key element for the development of their role as reading mediators in their teaching endeavors, as they will need to read with others and for others.

Furthermore, it is noteworthy that despite finding enjoyment in the activity, a utilitarian perspective of this construct prevails among them. Upon analyzing the interviews, no mentions were found that could account for the social dimension of reading, understood as a practice that enables them to become part of the reading community (Margallo, 2012). Instead, reading seems to occur in their individual space. Moreover, encounters with books are often sporadic. While they express enjoyment of reading and, consequently, the aesthetic experience that literary works provides them with, their reading activity is predominantly confined to academic settings due to the demands of their studies. Therefore, it could be stated that, during their pursuit of a degree in Language and Literature Pedagogy, their reading serves primarily as a means of acquiring educational knowledge for their future teaching practices.

Lastly, it is important to acknowledge the limitations of this study, which are primarily related to the data collection instrument. As it was an interview, participants primarily drew upon their spontaneous experiences and memories to respond. This may, to some extent, affect how they articulate their thoughts and beliefs about reading. Similarly, it is crucial to continue prioritizing the reading processes of future educators, aiming to enhance their relationship with reading and ultimately solidify their reading habits by the conclusion of their educational journey.

## Data availability statement

The original contributions presented in the study are included in the article/supplementary material, further inquiries can be directed to the corresponding author.

## Ethics statement

The studies involving humans were approved by Bioethics and Biosafety Committee of Pontifical Catholic University of Valparaíso (code: BIOPUCV-H486-2022). The studies were conducted in accordance with the local legislation and institutional requirements. The participants provided their written informed consent to participate in this study. Written informed consent was obtained from the individual(s) for the publication of any potentially identifiable images or data included in this article.

## Author contributions

CG: Conceptualization, Investigation, Methodology, Writing – original draft, Writing – review & editing. EP: Conceptualization, Investigation, Methodology, Writing – original draft, Writing – review & editing.
